# circRPPH1_025 Overexpression Promotes Migration and Invasion of Glioblastoma Multiforme

**DOI:** 10.1155/2022/4764028

**Published:** 2022-07-26

**Authors:** Lixiong Xue, Huahui Chen, Xiaolong Wang, Li Han, Yifan Liu, Xinmin Ding

**Affiliations:** ^1^Department of Neurosurgery, Shanxi Bethune Hospital, Shanxi Academy of Medical Sciences, Tongji Shanxi Hospital, Third Hospital of Shanxi Medical University, Taiyuan 030032, China; ^2^Department of Neurosurgery, Tongji Hospital, Tongji Medical College, Huazhong University of Science and Technology, Wuhan 430030, China

## Abstract

**Objective:**

To study the effect of circ_0000512 (circRPPH1_025) on the tumorigensis and development of glioblastoma and its molecular mechanism.

**Methods:**

The expression levels of circ_0000512 in normal astrocytes (NHA) and human glioblastoma cell lines (U87, U251, and A172) and the expression levels of circ_0000512 and linear RNA RPPH1 in U87 cells after RNase R treatment were detected by qRT-PCR. The effects of circ_0000512 knockdown or overexpression on the proliferation, migration, invasion, and epithelial-mesenchymal transition of U87 cells were detected by CCK-8 assay, cell colony formation assay, transwell invasion assay, wound healing assay, and western blot.

**Results:**

The expression of circ_0000512 was upregulated in glioblastoma cells, and the overexpression of circ_0000512 was beneficial to the proliferation, migration, invasion, and epithelial-mesenchymal transition of U87 cells, while knockdown of circ_0000512 showed the opposite results.

**Conclusion:**

circ_0000512 can be used as a potential target for early diagnosis and targeted therapy of glioblastoma multiforme.

## 1. Introduction

Glioblastoma, also known as glioblastoma multiforme (GBM), is the most common type of brain tumor in adults [1], accounting for approximately 17% of primary brain tumors [2]. GBM cells are often prone to infiltrative growth and invade surrounding normal brain tissues, so GBM is considered to be the most malignant type of glioma [3], and this aggressive phenotype is an important cause of GBM treatment failure and postoperative recurrence and metastasis [4]. Despite great advances in intensive treatment with surgery, radiotherapy, and chemotherapy, the prognosis of patients with malignant glioma remains poor, with a 5-year survival rate of 5% [5]. Early diagnosis and improving prognosis are key to the correct treatment of GBM. However, considering the poor effect of traditional treatment, it is urgent to find a reliable therapeutic target for malignant glioma.

Circular RNA (circRNA) is a new class of endogenous noncoding RNA discovered in recent years, whose circular structures composed of intron splicing are characterized by high stability, conservation, and specific expression [6, 7]. circRNAs are mainly located in the cytoplasm and regulate gene expression at the posttranscriptional level by adsorbing microRNA (miRNA), regulating the function of target miRNA [8]. They are involved in tumorigensis and development of various diseases [9–13]. For example, upregulation of circ_01844 can induce apoptosis of GBM and inhibit GBM cell proliferation and migration [14]. Circ-EPB41L5 can inhibit GBM tumorigenicity by sponging miR-19a to regulate the host gene EPB41L5 [15]. Therefore, circRNA is one of the potential targets for GBM diagnosis and treatment. Exploring the role of circRNAs in GBM may provide new ideas for the treatment of the disease.

circ_0000512 is a newly discovered circRNA, and Wang et al. [16] found that knockdown of circ_0000512 inhibited cell proliferation and promotes apoptosis in colorectal cancer by regulating miR-296-5p/RUNX1 axis, and circ_0000512 could be a potential target for colorectal cancer, but its mechanism in GBM is still unclear. Therefore, the aim of this study was to clarify the differential expression of circ_0000512 in GBM and reveal its impact on malignant biological behavior of GBM cells through a series of molecular biological techniques, laying a molecular biological foundation for targeted therapy.

## 2. Materials and Methods

### 2.1. Cell Culture

A human normal astroglial cell line (NHA) and three human glioblastoma cell lines (U87, U251, and A172) were obtained from the Cell Bank of the Chinese Academy of Sciences (Shanghai, China). All cell lines were grown in DMEM medium (Gibco, USA) containing 10% fetal bovine serum (FBS, Gibco, USA) and 1% penicillin-streptomycin at 37°C in a humidified atmosphere with 5% CO_2_.

### 2.2. Cell Transfection

The circ_0000512 overexpression plasmids (circ_0000512) and its negative control (vector) and the circ_0000512 knockdown plasmids (si-circ_0000512) and its negative control (si-NC) were designed and provided by Ribobio (Guangzhou, China). Then, according to the manufacturer' s instructions, LipofectamineTM 2000 (Invitrogen, USA) was utilized to transfect into U87 cells with the above plasmids. After 48 h, cells were collected for subsequent experiments.

### 2.3. Ribonuclease (RNase) R Treatment

Ribonuclease (RNase) R assay was used to evaluate the stability of circ_0000512 in U87 cells. RNase R (3 U/*μ*g) (Epicentre Technologies, USA) was added or not added to total RNA (2 *μ*g) and incubated at 37°C for 30 minutes. Then, the expression levels of circ_0000512 and RPPH1 were detected by qRT-PCR [17].

### 2.4. Quantitative Real-Time Polymerase Chain Reaction (qRT-PCR)

Total RNA from U87 cells was extracted using the Total RNA reagent (Thermo Fisher Scientific, Inc.) according to the manufacturer' s instructions. Then, RNA was reverse transcribed into cDNA using PrimeScript RTMaster Mix (Takala, Japan). Next, qRT-PCR was performed using the SYBR PremixEx Taq II kit (Takala, Japan) to detect the expression of RPPH1 mRNA and circ_0000512 in U87 cells, and GAPDH was used as an internal reference gene. PCR amplification procedure is as follows: 95°C, 1 min; 35 cycle of 95°C for 40 s, 58°C for 40 s, and 72°C for 45 s; and 72°C, 10 min. Genes' relative expression was calculated by 2^-*ΔΔ*Ct^ method [18].  Table 1 lists the primer sequences.

### 2.5. Cell Counting Kit-8 (CCK-8)

Transfected U87 cells (1 × 10^3^ cells/well) were seeded into 96-well plates for culture. After 24 h, the old medium was removed, and then, 10 *μ*L of cell counting kit-8 (CCK-8) reagent (Solarbio) and 90 *μ*L of DMEM were added to each well and incubated for 2 h. The viability of cells was detected by measuring the absorbance at 450 nm using a microplate reader (BioTek Instruments, USA) [19].

### 2.6. Colony Formation Experiments

Transfected U87 cells (5 × 10^2^ cells/well) were seeded into 6-well plates, and the medium was renewed every 3 days. After 14 days of culture, removing the previous medium, cells were fixed with 4% paraformaldehyde, and then, cells were stained with 0.1% crystal violet. Cell colonies > 50 cells were counted under the microscope, and the colony formation rate was calculated [20].

### 2.7. Wound Healing Experiment

The transfected U87 cells (5 × 10^5^ cells/mL) were seeded into 6-well plates, and when the cell growth reached 90%, a 200 *μ*L sterile pipette tip was used for wound scratching, and the cells were washed with PBS, then placed in a 6-well plate, and cultured in serum-free medium. Images at 0 h and 24 h postscratch were taken using an inverted microscope. The total wound area was measured using the ImageJ software, and the relative mobility was calculated [21].

### 2.8. Transwell Invasion Assay

The transfected cells (5 × 10^5^ cells/mL) were suspended in 200 *μ*L of serum-free medium, seeded in the upper chamber coated with matrigel (Corning, United States), and then added 600 *μ*L medium containing 10% FBS to the lower chamber. After 48 h of culture, the matrigel and cells on the upper surface of the membrane were wiped off with a cotton swab. The invading cells on the lower membrane surface were fixed with 4% paraformaldehyde, and then, the cells were stained with 0.1% crystal violet. The number of invasive cells was photographed and counted using an inverted microscope [22].

### 2.9. Western Blot

RIPA lysis buffer (Beyotime) was used to extract cellular proteins, and BCA protein detection kit (Beyotime) was used to detect the concentration of protein samples. Then, the samples were transferred to PVDF membrane by SDS-PAGE, and after blocking with 5% skim milk solution for 1 h, the membranes were hatched together with the primary antibodies against E-cadherin (1 : 10000, ab40772; Abcam), N-cadherin (1 : 5000, ab76011; Abcam), Snail (1 : 1000, ab216347; Abcam), and GAPDH (1 : 2500, ab9485; Abcam) overnight at 4°C; then, the membranes were incubated with secondary antibodies (1 : 10000, ab205718; Abcam) for 1 h at room temperature. Finally, protein signals were detected using enhanced chemiluminescence reagent (Beyotime), and semiquantitative analysis was performed by the Image Pro Plus 6.0 software (Media Control, Inc.).

### 2.10. Statistical Analysis

The experimental data were statistically analyzed using the SPSS 22.0 software. *T*-test was used for comparison between the two groups, and one-way analysis of variance for comparison among multiple groups. The results were presented as mean ± standard deviation (SD), and all tests were independently repeated three times, and *P* < 0.05 was considered statistically significant.

## 3. Results

### 3.1. circ_0000512 Is Upregulated in GBM Cells

**Figure 1:**
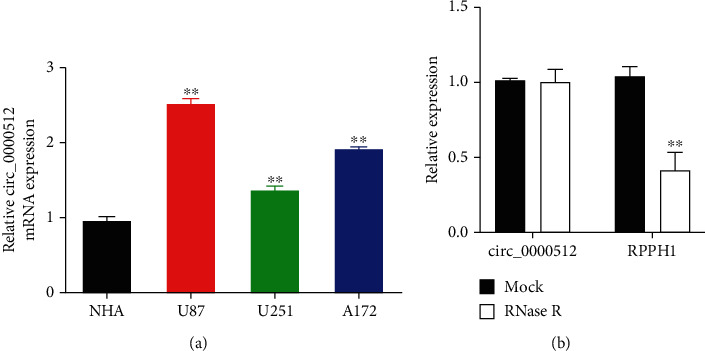
Expression of circ_0000512 in GBM cells. (a) The expression levels of circ_0000512 in the cells of each group were detected by qRT-PCR, ^∗∗^*P* < 0.01 vs. NHA group; (b) qRT-PCR was used to detect the expression levels of circ_0000512 and linear RNARPPH1 in U87 cells after RNase R treatment, ^∗∗^*P* < 0.01 vs. Mock group.

To clarify whether circ_0000512 is involved in the occurrence of GBM, we examined the expression of circ_0000512 in GBM cell lines. The results showed that the expression level of circ_0000512 in GBM cell line was significantly higher than that in NHA, and the U87 cell line with the highest expression level of circ_0000512 will be used for subsequent experimental studies (Figure 1(a)). First, we examined the stability of circ_0000512 in U87 cells. The results showed that circ_0000512 was resistant to RNase R (Figure 1(b)), indicating that circ_0000512 was not easily degraded in U87 cells. These results suggest that circ_0000512 expression upregulation may be involved in the tumorigenesis and progression of GBM.

### 3.2. circ_0000512 Overexpression Promotes the Proliferation, Invasion, and Migration of U87 Cells

**Figure 2:**
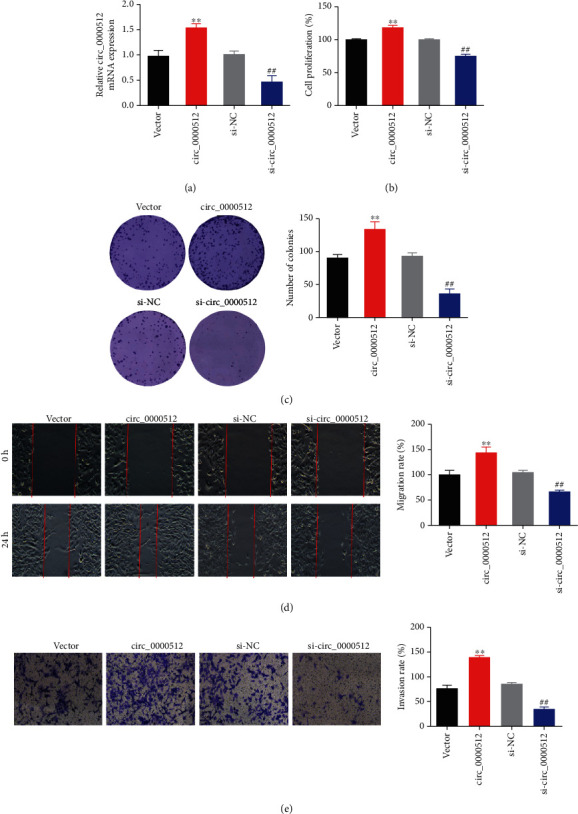
The effect of circ_0000512 on the proliferation, migration, and invasion of U87 cells. (a) qRT-PCR was used to detect the transfection efficiency of circ_0000512 knockdown or overexpression U87 cells; (b) CCK-8 method was used to analyze the proliferation ability of circ_0000512 knockdown or overexpression U87 cells; (c) cell colony formation experiments were used to analyze the proliferation ability of circ_0000512 knockdown or overexpression U87 cells; (d) wound healing experiments were used to evaluate the migration ability of circ_0000512 knockdown or overexpression U87 cells; (e) transwell assay was used to analyze the invasive ability of circ_0000512 knockdown or overexpression U87 cells. ^∗∗^*P* < 0.01 vs. vector group, ^##^*P* < 0.01 vs. si-NC group.

To investigate the role of circ_0000512 in GBM, we constructed knockdown and overexpressed circ_0000512 plasmids and transfected the plasmids into U87 cells, and qRT-PCR was used to verify the transfection efficiency (*P* < 0.01) (Figure 2(a)). Then, the proliferation ability of U87 cells was detected by CCK-8 method and colony formation assay. The results showed that overexpression of circ_0000512 could significantly promote cell proliferation and cell colony formation (*P* < 0.01) (Figures 2(b) and 2(c)). In addition, we also performed wound-healing assays and transwell analyses to determine the role of circ_0000512 in GBM invasion and metastasis. The results showed that overexpression of circ_0000512 significantly enhanced the migration and invasion abilities of U87 cells (*P* < 0.01) (Figures 2(d) and 2(e)). However, U87 cells knocked down circ_0000512 showed the opposite result. Collectively, the above data suggest that circ_0000512 overexpression contributes to the proliferation, migration, and invasion of U87 cells.

### 3.3. circ_0000512 Overexpression Promotes Epithelial Mesenchymal Transformation of U87 Cells

**Figure 3:**
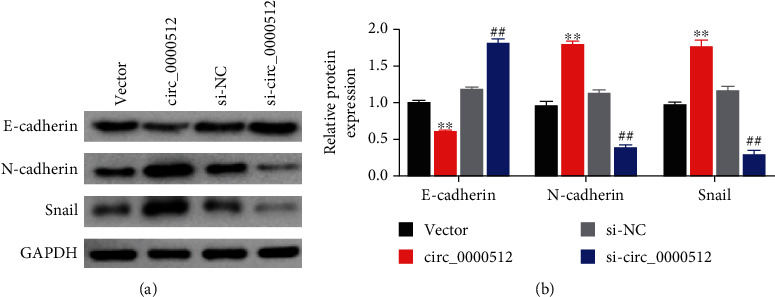
The effect of circ_0000512 on EMT of U87 cells. (a) Western blot was used to detect the protein levels of E-cadherin, N-cadherin, and Snail in circ_0000512 knockdown or overexpressed U87 cells. (b) Quantification of the western blot results from three independent experiments. ^∗∗^*P* < 0.01 vs. vector group, ^##^*P* < 0.01 vs. si-NC group.

Next, we further clarified the migration and invasion roles of circ_0000512 in glioma blasts by detecting the expression of epithelial mesenchymal transformation- (EMT-) related proteins. The results showed that compared with the control group, circ_0000512 overexpression significantly increased the protein expression of N-cadherin and Snail in U87 cells and decreased the protein expression of E-cadherin, while knockout of circ_0000512 had the opposite effect (*P* < 0.01) (Figures 3(a)–3(b)). It indicated that the overexpression of circ_0000512 was beneficial to the migration and invasion of glioma blasts.

## 4. Discussion

GBM has the highest incidence and malignancy in adult primary brain tumors, and the life cycle of GBM patients is only 12 to 15 months [23]. Meanwhile, as GBM exhibits highly invasive growth, the outcome remains poor. With development of high-throughput sequencing technology, increasing noncoding RNAs are found to be abnormally expressed in tumors. There are also studies on circRNAs in GBM. Zhu et al. [24] first identified 1411 differentially expressed circRNAs in GBM tissues. Xia et al. [25] further found that circ-AKT3 is downregulated in GBM tissues and inhibits the proliferation ability, radiation resistance, and in vivo tumorigenicity of GBM cells. As such, as reported by Zhu et al., circ_0001946 is lowly expressed in GBM cells and suppresses GBM cell proliferation, migration, and invasion by inhibiting miR-671-5p expression [26]. Studies have shown that circMMP9, which is upregulated in GBM tissues, acts as a sponge for miR-124 on CDK4 and AURKA target genes regulating the expression of miR-124, thereby participating in the proliferation, invasion, and metastasis of GBM cells [27]. Therefore, circRNAs may be involved in the biological process of GBM in a variety of ways and can be used as biological markers to suggest the disease process of GBM in these processes. In this study, circ_0000512 was found to be upregulated in GBM cells, and its overexpression promoted the proliferation, invasion, and migration of U87 cells, while silencing circ_0000512 inhibited the malignant behaviors of cancer cells. Our data suggests that circ_0000512 is also involved in the malignant process of GBM cells.

EMT is considered as one of the important mechanisms of malignant process, invasion, and metastasis of glioma cells. It mainly manifests as the loss of epithelial cell morphology of the cell, loss of cell polarity, disappearance of tight junctions, decrease of intercellular adhesion, and morphological transformation to stromal cells with invasive and metastatic ability, as the process is very critical to the process of tumor invasion and metastasis [28]. Studies have shown that during EMT in tumors, E-cadherin converts to N-cadherin, as shown by downregulation of E-cadherin expression, while epithelial marker N-cadherin and mesenchymal marker Vimentin are upregulated [29] and their expression changes are one of the basic markers of EMT development [30]. Glioma patients with high expression of Vimentin and Snail have a worse prognosis [31, 32]. In addition, it has also been found that knockdown of Snail1 in glioma cells in vitro weakens the proliferation, invasion, and migration ability of GBM cells by reducing Vimentin and increasing E-cadherin expression [32, 33]. In this study, we found that when circ_0000512 was overexpressed, the protein levels of E-cadherin were significantly decreased (*P* < 0.05) and N-cadherin and Snail were significantly increased in cells, which subsequently promoted occurrence of EMT in GBM cells; however, silencing circ_0000512 inhibited the process of EMT. These results suggest that circ_0000512 may aggravate GBM progression by promoting EMT, migration, and invasion.

## 5. Conclusion

In conclusion, the upregulation of circ_0000512 is beneficial to the occurrence of EMT, migration, and invasion of glioblastoma cells and promotes the tumorigenesis and development of GBM. It is suggested that circ_0000512 can be used as a potential target for early diagnosis and targeted therapy of glioblastoma.

## Figures and Tables

**Table 1:** Primer sequence.

Gene name	Primer sequence
circ_0000512	F 5′-GGAACAGACTCACGGCCA-3′
	R 5′-CATCTCCTGCCCAGTCTGAC-3′
RPPH1	F 5′-GAGCTGAGTGCGTCCTGTC-3′
	R 5′-TCAGGGAGAGCCCTGTTAGG-3′
GAPDH	F 5′-CGCTCTCTGCTCCTCCTGTTC-3′
	R 5′-ATCCGTTGACTCCGACCTTCAC-3′

## Data Availability

The data used to support the findings of this study are available from the corresponding author upon request.
